# *Bacillus* for Plant Growth Promotion and Stress Resilience: What Have We Learned?

**DOI:** 10.3390/plants11192482

**Published:** 2022-09-22

**Authors:** Teboho Tsotetsi, Lerato Nephali, Motumiseng Malebe, Fidele Tugizimana

**Affiliations:** 1 Department of Biochemistry, University of Johannesburg, Auckland Park, Johannesburg 2006, South Africa; 2 International R&D Division, Omnia Nutriology, Omnia Group (Pty) Ltd., Johannesburg 2021, South Africa

**Keywords:** *Bacillus*, biostimulant, plant growth, stress resilience, metabolomics

## Abstract

The rhizosphere is a thin film of soil that surrounds plant roots and the primary location of nutrient uptake, and is where important physiological, chemical, and biological activities are occurring. Many microbes invade the rhizosphere and have the capacity to promote plant growth and health. *Bacillus* spp. is the most prominent plant growth promoting rhizobacteria due to its ability to form long-lived, stress-tolerant spores. *Bacillus*-plant interactions are driven by chemical languages constructed by a wide spectrum of metabolites and lead to enhanced plant growth and defenses. Thus, this review is a synthesis and a critical assessment of the current literature on the application of *Bacillus* spp. in agriculture, highlighting gaps that remain to be explored to improve and expand on the *Bacillus*-based biostimulants. Furthermore, we suggest that *omics* sciences, with a focus on metabolomics, offer unique opportunities to illuminate the chemical intercommunications between *Bacillus* and plants, to elucidate biochemical and molecular details on modes of action of *Bacillus*-based formulations, to generate more actionable insights on cellular and molecular events that explain the *Bacillus*-induced growth promotion and stress resilience in plants.

## 1. Introduction

Edaphic factors and genetics play a pivotal role in the growth and yield of crop plants [1]. Over the past decades, genetic engineering and plant breeding approaches have been employed to develop new cultivars with desired traits, such as high yield and resistance to environmental stresses [2]. However, there is a less commercial success for genetically modified crops due to ethical constraints concerning genetically modified organisms (GMO) [3]. To obtain better crop yield, applications of chemical fertilizers have been the opted strategy. However, over time, studies and empirical evidence have shown that this traditional method—the use of chemical fertilizers—is not sustainable due to the inherent negative effects these products have on the environment. The excessive utilization of chemical fertilizers has shown to lead to toxic build-up of heavy metals, soil acidification and soil crust, thereby reducing the soil content of organic matter and humic substance. Soil acidification reduces crop phosphate intake, raises the concentration of harmful ions in the soil, and inhibits crop growth [4].

The incorporation of biostimulants, such as plant growth-promoting rhizobacteria (PGPR)-based formulations, in cropping systems has increasingly shown to be a promising strategy for sustainable agriculture and global food security, aligning with the United Nations sustainable development goals (SDGs) [5]. A broad array of bacterial species has been reported to possess plant growth-promoting attributes with the prominent species belonging to the genus *Bacillus*. Members of the genus *Bacillus* are ubiquitous, Gram-positive, and aerobic bacteria [6,7]. *Bacillus* species produce a multitude of enzymes, antibiotics, and metabolites which give them prominent applications in various sectors such as pharmaceuticals and agriculture. Furthermore, their uniqueness and popularity arise from their spore forming ability which enables them to grow in unconducive environmental conditions [8]. When applied to the plant as dormant cells, these *Bacillus* spores must germinate to form metabolically active cells. Following germination, these bacteria could be attracted by chemotaxis and leading to the root colonization process (which is mechanistically complex) and exerting growth promotion potentials [9]. Upon colonization, *Bacillus* spp. elicit direct (e.g., siderophore production, nitrogen fixation, phytohormone production and nutrient solubilization) and indirect mechanisms (such as production of exo-polysaccharides (EPS), biofilm formation, hydrogen cyanide (HCN) and lytic enzymes) to promote plant growth and yield, under various environmental conditions [7].

Thus, this review is a synthesis and a critical assessment of the current literature on the application of *Bacillus* spp. in agriculture, highlighting gaps that remain to be explored to improve and expand on the *Bacillus*-based biostimulants that are currently on the market. Considering ongoing efforts to understand the chemical intercommunication between rhizobacteria and plants, we herein review the current knowledgebase on key metabolites secreted by both the bacteria and the plant, their role in the communication between the two organisms, and responses to various environmental stresses. We acknowledge the growing literature and reports on emerging studies and insights on molecular mechanisms underlying rhizobacteria–plant interactions. The intent of this review is to provide a synthesis on the belowground chemical lexicon used for these interactions, with a focus on *Bacillus*, briefly summarizing the current state of knowledge in this regard. Furthermore, we discuss and emphasize that *omics* sciences, with a focus on metabolomics, offer unique opportunities to decode the “dark matter” in the chemical intercommunications between *Bacillus* and plants, to elucidate biochemical and molecular details on modes of action of *Bacillus*-based formulations, to generate more actionable insights on cellular and molecular events that explain the *Bacillus*-induced growth promotion and stress resilience in plants.

## 2. *Bacillus* spp.-Based Biostimulants and Growth Promotion Mechanisms

Various plant-associated *Bacillus* spp. have been commercialized as biostimulants for plant protection and growth promotion [5]; and examples of these formulations are given in Table 1. However, there has been a slow rate of exploitation of these *Bacillus*-based formulations. This is mainly due to a lack of understanding of the chemistry and biochemical mechanisms underlying the modes of action of the *Bacillus* spp. strains and defining the efficacy of these formulations when applied to crop plants under field conditions. The latter are multifactorial, impacting the growth and productivity of the plant. Various studies have illustrated discrepancies in the performance of rhizobacteria applied under well-controlled conditions and field conditions [10]. There is indeed a need for fundamental studies to understand the metabolism and the biochemistry of *Bacillus* strains, to elucidate biochemical and molecular events that define *Bacillus*-plant interactions at different levels and in both systems, i.e., bacteria and host plants.

**Figure 1:**
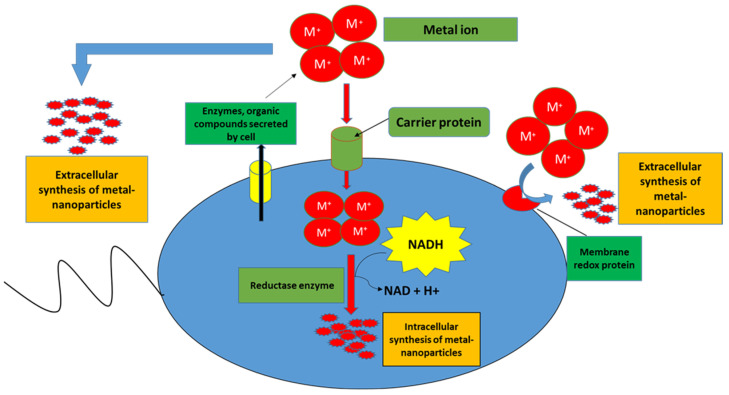
Intra-and extracellular biosynthesis of metal-nanoparticles by *Bacillus* spp. Extracellular biosynthesis of metal-nanoparticles is carried out by trapping metal ions on the cell surface and reducing them in the presence of secreted enzymes or metabolite and/or membrane-bound enzymes. In the intracellular biosynthesis of metal-nanoparticles, after transfer of metal ions into cell cytoplasm, the metal ions are reduced as a result of metabolic reactions with enzymes such as alpha-NADPH-dependent nitrate reductase.

Despite the lack of fundamental understanding of molecular mechanisms that govern modes of action of *Bacillus* spp., the applications of *Bacillus*-based formulations on plants (Table 1) have shown positive impacts such as plant growth promotion. The latter is thought to be due to *Bacillus*-enhanced nutrient uptake and hormonal modulations. Nutrient availability plays a pivotal role in seed germination and plant growth. However, the bioavailable forms of nutrients such as phosphorus and nitrogen are limited in the rhizosphere. Thus, *Bacillus* spp. assists in converting the complex forms of these essential nutrients to simple available forms [2]. *Bacillus* spp. can facilitate the uptake of phosphates by the roots through the secretion of phosphatases and low molecular weight organic acids such as formic acid, acetic acid, lactic acid, glycolic acid, fumaric acid, and succinic acid which acidify the environment to aid the conversion of inorganic phosphates into free phosphate [22]. In addition, *Bacillus* spp. also produces siderophores which help to solubilize iron from minerals and organic compounds in the rhizospheres. Low molecular weight siderophores such as enterobactin, pyochelin, alcaligin, and rhizoferin have iron-chelating ability making it very arduous for other microbes to access iron. The siderophore–iron complexes can be readily absorbed by the plants; however the transport systems involved therein are not fully understood [23]. *Bacillus* also facilitates iron mobility by inducing the upregulation of iron acquisition genes in plants. Additionally, studies have shown that *Bacillus* can increase the concentration of metal ions, which are often a limiting factor for plant growth by breaking them down into nanoparticles thus facilitating their mobilization as illustrated in Figure 1 [24].

Furthermore, upon colonization, *Bacillus* can directly enhance plant growth by the secretion of cytokine hormones and volatile organic compounds (VOCs) that modify plant hormone networks, promoting cell division and growth [25]. Two VOCs, 3-hydroxy-2-butanone and 2,3-butanediol, which are produced by *B. subtilis*, have shown to boost plant growth by altering cytokinin and ethylene homeostasis [26]. In another study by Zhang et al. [27], *B. subtilis* modulated auxin homeostasis by lowering its levels in leaves and inducing optimal growth in the *Arabidopsis* plant. Spermidine, which is also produced by various *Bacillus* spp., was reported to promote plant growth via the induction of expansins, and the reduction of ethylene levels in plants [28]. Despite the proposed models that explain these plant growth promotion events induced by *Bacillus* spp., the chemical language that dictates, mechanistically, *Bacillus*-plant interactions is still poorly understood. Thus, in the following section, we look at the growing literature on chemical communications between plants and PGPR, pointing out key postulations.

## 3. Chemical Conversation between Plant and *Bacillus* spp. Which Leads to Plant Growth Promotion

**Figure 2:**
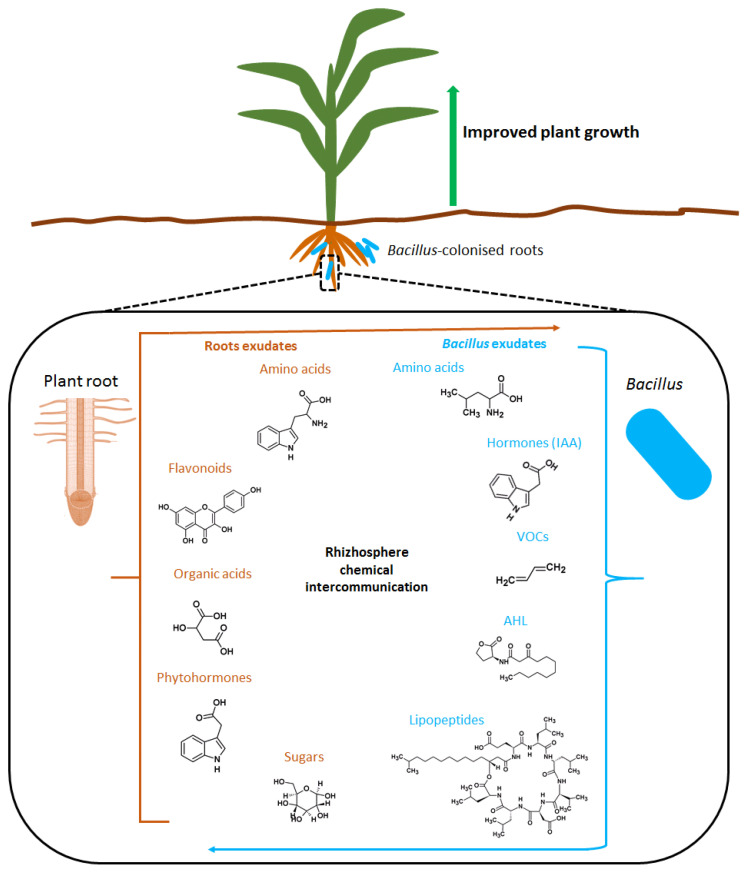
Some molecules involved in *Bacillus*-plant interactions. Plants produce various compounds including the hormones salicylic acid (SA), jasmonic acid (JA), cytokinins (CK), and indole acetic acid. Plants also produce organic acids, flavonoids, amino acids, and 1-aminocyclopropane-1-carboxylate (ACC) as signaling molecules. On the other hand, *Bacillus* produce volatiles such as 2,3 butanediol, lipopeptides such as surfactin, and phytohormones such as gibberellic acid and acyl homoserine lactone (AHL) as signaling molecules.

The communication between plant roots and microbes in the rhizosphere is highly organized and regulated through a dynamic range of specialized metabolites and exudates which ultimately result in altered gene expression in one or both of the interacting partners [29]. The compounds produced by *Bacillus* spp. include phytohormones, ACC deaminase, volatile organic compounds, polyamines, lipopeptides, and acyl homoserine lactose. On the other hand, plants produce a range of various low-molecular weight molecules such as phytohormones, organic acids, and flavonoids (Figure 2).

The success of microbial based biostimulants relies on the root colonization ability of the beneficial microbes. As abovementioned, root colonization is a complex process which involves a highly dynamic molecular language (which remains enigmatic) between the microbes and the plant in the rhizosphere. Furthermore, this process is influenced by various parameters such as bacterial communities, root architecture and exudates (which depend on the developmental stages and physiological statuses of the plants), (a)biotic factors, and the physicochemical and biological components of the soil [30]. An elaboration on these belowground interactome associates is beyond the scope of this review; however, it suffices here to highlight that understanding the various factors that define the belowground interactions between the bacteria and plant is essential for better and novel exploration of these beneficial bacteria for agricultural practices.

### 3.1. The Chemical Lexicon of Bacillus spp.-Phytohormones and Organic Compounds

*Bacillus* spp. are known to produce a wide range of hormones that act as signal molecules in the rhizosphere and involved in the belowground interaction networks (Figure 2). Some of the phytohormones known to be produced by *Bacillus* spp. include indole acetic acid (IAA), cytokinins (CKs), gibberellins (GAs), and abscisic acid (ABA) [31,32]. IAA is a potent signaling molecule vital for plant-microbe interactions and directly improve plant growth by elevating plant auxin pool which leads to cell elongation, vascular tissue development, and apical dominance [32]. Recent metabolomics and molecular studies have enabled the identification of genes involved in IAA biosynthesis in *B. amyloliquefaciens* showing that it can occur through multiple pathways [33]. Among the various IAA synthesis pathways that have been identified, tryptophan has been confirmed to be the main precursor [32]. Furthermore, studies have pointed out that plant roots secrete tryptophan in the rhizosphere (Figure 2) which is utilized by rhizobacteria as a precursor for IAA biosynthesis [34,35]. This typically points to the belowground chemical interactomes that define *Bacillus*–plant interactions.

*Bacillus* spp. produce a vast range of cytokinins (CKs) (Figure 2) including zeatin, zeatin riboside, zeatin glycoside, izopentyl adenine, and izopentyl adenosine, which have been reported to be produced by *B. cereus*, *B. megaterium*, and *B. subtilis* [36]. Inoculation of lettuce plants with CK-producing *B. subtilis* increased shoots biomass [25]. Furthermore, the production of zeatin and zeatin riboside by *B. subtilis* can elicit the exudation of amino acids from the roots in wheat, subsequently increasing the diversity and quantity of beneficial microbiota in the rhizosphere [37]. Another group of phytohormones that is produced and secreted by *Bacillus* spp. is gibberellin (Figure 2) which is also involved in different plant developmental processes and the regulation of many physiological processes [38]. In the lettuce plant, the production of gibberellic acid by *Bacillus* spp. has been associated with an increase in nutritional metabolites such as amino acids, macro-and micronutrients, fructose, and carotenoids, thus increasing the quality of the crop [39].

In addition to hormones, rhizobacteria are reported to use volatile organic compounds (VOCs) in the chemical interactions with plants or other microorganisms (Table 2). Studies have shown that various genera of bacteria can regulate plant growth from a distance [40]. Some of these VOCs exuded by bacteria act as growth promoter molecules [41]. VOCs are low molecular weight and high vapor pressure compounds and act as signal molecules over short distances [42]. These features enable VOCs to facilitate intercellular and organismal interactions [43]. VOCs from *Bacillus* spp. are categorized into aldehydes, ketones, alkyls, alcohols, alkenes, esters, acids, ethers, heterocyclic, and phenolic compounds; however, many of these VOCs are still unknown [44,45]. Due to the structural diversity of these microbial VOCs, many natural functions have been inferred as highlighted in Table 2. For example, the production of the VOCs albuterol and 1,3-propanediol by *B. subtilis* induced differential expression of genes involved in the biosynthesis of the phytohormones auxin, gibberellin, cytokinin, and ethylene [42]. This subsequently leads to altered levels of the endogenous content of the related hormones in the roots and leaves, suggesting the involvement of these hormones in signal transduction pathways induced by VOCs which ultimately enhances plant growth. The natural function of these microbial VOCs is not only limited to plant growth but also infer protection against biotic stresses and induce plant tolerance against abiotic stresses. Investigations of these *Bacillus* VOCs-mediated effects on plants have focused on how they influence the signaling of phytohormones such as salicylic acid (SA), jasmonic acid (JA), ethylene, auxin, etc. [40]. Even though the effects of these *Bacillus* VOCs on plants are well documented, the underlying signaling mechanisms are still poorly understood [46].

As abovementioned, the belowground chemical interactome is highly complex and dynamic. One of the factors that define the performance of these PGPR is the efficacy in the root colonization by the bacteria, which is highly influenced by root exudates. The latter depends on the developmental stages and physiology of the plants. Furthermore, the microorganisms in the rhizosphere have diverse roles in supporting plant growth, development, and inhibition of host pathogens. This implies interdependency between the host and microbes in the aboveground and belowground interactions. Thus, understanding rhizosphere colonization mechanisms by PGPR is essential for generating inoculants able to compete and efficiently colonize the rhizosphere of plant crops and having a great impact on crop production and more consistent results.

### 3.2. Plant Root Exudates in Plant–Bacillus Crosstalk

Root exudates are a complex mixture of primary and secondary metabolites released into the rhizosphere by the roots and they maintain constant communication between plants and microbes in the rhizosphere [52]. Root exudates are composed of amino acids, organic acids, phenolics, sugars, and proteins with their highly diversified chemical properties based on different plant species, plant growth stages and the microbiota in the rhizosphere [53]. One of the roles of root exudates is to serve as signals for root colonization by beneficial microbes [54] as shown in Figure 2. PGPR can colonize the roots through chemotaxis, which is the mobility capacity of the bacteria along a chemical signal gradient [55]. Thus, root exudates are the architects of the rhizosphere and congruently the colonization of bacteria on the roots can modulate the metabolites exudation pattern [56].

The bacterial chemotaxis is elicited when a root exudate molecule binds to its cognate receptor [57]. Given the chemical diversity of root exudates, systematic identification of chemo-attractants and chemo-repellents in root exudates and elucidation of how these various root-secreted compounds are detected by multiple chemoreceptors of a PGPR strain can provide a comprehensive understanding of how PGPR colonizes the rhizosphere. In a study by Feng et al. [57], 39 chemo-attractants and 5 chemo-repellents were identified from cucumber root exudates for a well-studied PGPR strain *B. amyloliquefaciens* SQR9. In this study a mutant strain with 8 putative chemoreceptors deleted, lost chemotactic response to all the 44 compounds showing the importance of these chemoreceptors in plant–PGPR interactions and subsequent colonization. Further characterization of these chemo-effectors (root exudates) and chemoreceptors will broaden our insights into *Bacillus*–root interaction and provide valuable information to enhance the rhizosphere colonization ability of *Bacillus* spp., which will promote their application in agricultural production.

Post root colonization by *Bacillus*, the bacteria proliferate by receiving key signaling compounds and nutrients from the root exudates which ultimately lead to biofilm formulation on the root system which is an indication of a successful root colonization [58]. A study by Yuan et al. [58] showed that root exudates of banana containing organic acids induced both chemotaxis and biofilm formation in *B. amyloliquefaciens.* In this study, malic acid showed the greatest chemotactic response whereas fumaric acid significantly induced biofilm formation. Root exudates can be modified by environmental perturbations and thus influence the plant–PGPR interaction as it was illustrated in a study by Cesari et al. [59] where drought conditions modified the pattern of molecules exuded by roots, increasing the exudation of naringenin, oleic, citric, and lactic acids, and stimulating the release of terpenes of known antioxidant and antimicrobial activity. Changes in the molecular profiles of these exudates due to drought allowed for enhanced interaction between the roots and PGPR thus reversing the negative effects of drought condition on plant growth. These findings can assist in the formulation of biostimulant inoculants containing key molecules exuded during stress which can improve plant–PGPR interactions and thus promote plant growth and enhance defenses against various stresses.

## 4. *Bacillus* spp. Confers Protection to the Plant from Environmental Stresses

Major yield deficiencies, crop damage, and changes in growth rates of plants are caused by abiotic and biotic stresses. Many strategies have been proposed to combat the negative impacts of environmental stresses, and these have included, for instance, the overexpression of single genes encoding enzymes involved in the transportation of ions and scavenging of reactive oxygen species (ROS) being the most popular over the years. The application of this approach is limited due to the resultant pleiotropic effects on the growth of the plant and multiple pathways involved in response to environmental stress [60]. Another strategy is the use of chemical-based fertilizers and pesticides which are increasingly becoming harmful to the environment and some of these xenobiotics have become highly toxic in the agro-food chain. Therefore, nonharmful, environmentally friendly, sustainable, and nature-inspired solutions and strategies are urgently needed, and these include the use of PGPR formulations. The latter can aid in alleviating the effects of environmental stress. Several studies have demonstrated that *Bacillus* spp.-based biostimulants trigger plant protective mechanisms against both abiotic and biotic stresses.

### 4.1. Bacillus spp. against Biotic Stress

**Figure 3:**
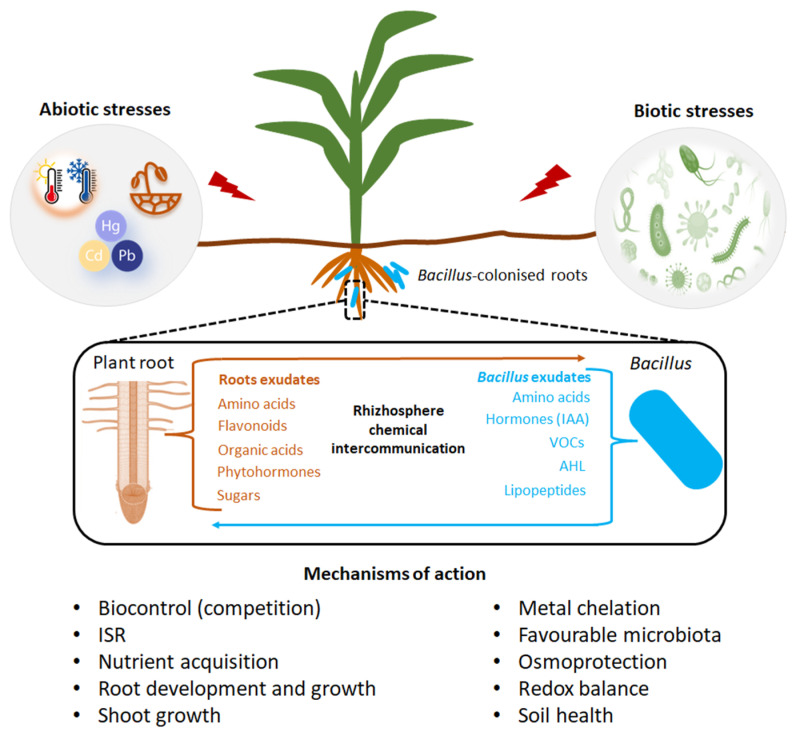
An overview of mechanisms employed by *Bacillus* spp. in the mitigation of biotic and abiotic stresses. *Bacillus* produce cyclic lipopetides which activate pathways regulated by jasmonic acid and ethylene and thus elicit induced systemic resistance (ISR). They also produce hydrogen cyanide (HCN) and the enzyme 1-aminocyclopropane-1-carboxylate (ACC) deaminase which lower the levels of plant ethylene. Siderophores produced by *Bacillus* chelate iron thus making it unavailable to the pathogen. Several root associated *Bacillus* spp. bacteria produce zeatin, gibberellic acid (GA), indole-3-acetic acid (IAA), salicylic acid (SA), abscisic acid (ABA) as well as volatile organic acids (VOCs) which help plants to withstand stress by enhancing its antioxidant potential, by up-regulation of the antioxidant system and by accumulation of compatible osmolytes thus reducing oxidative stress-induced damage; improving photosynthetic capacity and membrane stability; promoting cell division and stomatal regulation; stimulating growth of root system, and acquisition of water and nutrients.

Biological control, utilizing beneficial microbes, is an excellent approach to limiting the adverse effect of disease-causing microbes on plant health and productivity. Considerable effort has been placed on identifying microbial biocontrol agents that can repress phytopathogens, especially those that are responsible for soil-borne diseases, and that can enhance agricultural productivity [61,62]. Many strains of *Bacillus* exhibit the ability to act as biocontrol agents against pathogens and thus can be used to suppress diseases [63]. Several mechanisms, both direct and indirect, are responsible for their ability to control pathogenic microbes as shown in Figure 3. These include the production of a wide array of antibiotic compounds (lipopeptides), the ability to form endospores, the ability to form biofilms on root surfaces, and the ability to induce host systemic host resistance, and stimulate plant growth [64].

Biotic stresses often lead to the production of reactive oxygen species (ROS) which lead to oxidative stress and are toxic to the cells. The inoculation of plants with *Bacillus*-based formulations has shown to elicit the production of antioxidant defense enzymes such as superoxide dismutase and peroxidase, which scavenge the ROS [65]. In a study by Zebelo et al. [66], cotton plants inoculated with *Bacillus* spp. demonstrated an increase in gossypol and jasmonic acid levels and secretion reducing larval feeding by *spodoptera exigua.* There was also up-regulation of genes involved in synthesis of allelochemicals and jasmonates in the inoculated plants. Furthermore, *Bacillus* spp. secrete various catabolic enzymes such as proteases, chitinases, and glucanases, as well as peptide antibiotics and secondary metabolites that contribute to pathogen suppression [40]. *Bacillus* spp. secrete cyclic lipopeptides such as iturin and surfactin (Figure 3) which play a role in disease suppression by acting as bi-functional molecules through their antifungal activity and elicitation of induced systemic resistance (ISR). The latter involves pathogen recognition at plant cell surface, stimulation of early cellular immune-related events, systemic signaling through a fine-tuned-hormonal cross talk and activation of defense mechanisms [67]. *Bacillus* spp. can elicit ISR in plants, which switches on pathogenesis related genes, mediated by phytohormone signaling pathways and defense regulatory proteins to pre-condition plants against future pathogen ambush [68].

*Bacillus* spp. secrete the secondary metabolites, cyclic lipopeptides (CLPs) which are involved in developmental processes such as motility and biofilm formation as well as biocontrol primarily based on their antimicrobial activity [69]. The synthesis of these CLPs is accomplished by multimodular peptide synthetases and depends on a functional phospho-pantheinyl transferase (Sfp) which transfers 4′-phosphopantetheine from coenzyme A to the carrier proteins during non-ribosomal synthesis [70]. Some CLPs secreted by *Bacillus* have emerged as plant immunity elicitors (Figure 3). The best-described *Bacillus* CLP is surfactin. When applied as pure compound on roots, surfactin induced ISR in bean, tomato, tobacco, against *B. cinerea*, in melon against *Podosphaera fusca*, and peanut, against *Sclerotium rolfsii* [71,72,73,74]. The *Bacillus* CLP iturin has shown high bioactivity when applied to leaves as compared to the roots. The biochemical and molecular mechanisms that lead to this contrast are not well understood [75].

*Bacillus* strains also produce volatile organic compounds which act as elicitors of plant immunity. The most characterized VOCs secreted by *Bacillus* are 2,3-butanediol and acetoin (Figure 3) produced from glucose in the central metabolism [76]. In a study by Rudrappa et al. [77], exogenous application of acetoin triggered ISR and protected the maize plant against *Pseudomonas syringae*. What is intriguing in this study is that the expression of acetolactate synthase (an enzyme involved in the synthesis of acetoin) was significantly upregulated in the presence of maize root exudates during the late exponential growth phase, suggesting that root exudates play a role in eliciting acetoin biosynthesis in *Bacillus* [78]. The effects exerted by these secondary metabolites produced by *Bacillus* on plant health still need further elucidation as the response of the plant in the tandem presence of the beneficial *Bacillus* and the pathogen is complex.

### 4.2. Bacillus spp. against Abiotic Stress

Being sessile organisms, plants have to withstand various adverse abiotic stresses such as drought, salinity, heat/or cold, and heavy metal toxicity which pose a major threat to agriculture by negatively impacting plant growth and yield worldwide [79]. These stresses elicit stress responses in plants, including an accumulation of reactive oxygen species (ROS) and reduced photosynthetic activity, which ultimately leads to reduced plant growth and crop yield. PGPR such as *Bacillus* spp. can mediate the induction of abiotic stress responses in plants [80]. These responses to abiotic stresses are attributed to metabolic regulations which often require wide changes in the concentration, composition, and distribution of both primary and secondary metabolites. Biostimulants containing *Bacillus* strains have shown the potential to stimulate abiotic stress tolerance [81]. However, the biochemical and molecular mechanisms governing this *Bacillus*-induced stress resistance and tolerance are still enigmatic. Mechanisms suggested including changes of phytohormone levels through the secretion of phytohormones by *Bacillus* or ACC deaminase activity that decrease ethylene levels [82].

Cytokinins such as zeatin produced by *Bacillus* play a pivotal role in maintaining cellular proliferation and differentiation [83]. A study by Hussain and Hasnain [84] showed that the extracts obtained from cell cultures of *B. licheniformis* and *B. subtilis* are capable of increasing the weight and size of cucumber cotyledons separated from the seedlings, by inducing plant cell division due to the presence of cytokinins zeatin and zeatin ribose thus enhancing tolerance to salt stress. Cytokinin production has also been related to the ability of *Bacillus* species, such as *B. subtilis*, to increase plant tolerance to drought in lettuce [85] and *Platycladus orientalis* [86], or against salinity in wheat, due to the production of Zeatin, promoting plant growth [87]. *Bacillus* also produces auxins such as indole-acetic acid (IAA) which has been reported to confer tolerance against heavy metals [87,88]. For example, the study by Sun et al. [88] demonstrated that the application of IAA-producing *Bacillus altitudinis* alleviates iron stress in *Triticum aestivum* L. seedlings by both bioleaching of iron and up-regulation of genes encoding ferritins. Another study by Ji et al. [87] also showed that wheat treatment with IAA-producing *B. subtilis* can increase plant tolerance under salinity conditions.

*Bacillus* is also capable of producing gibberellic acids (GAs) which is a group of phytohormones involved in the processes of seed germination, flower initiation, leaf expansion, stem elongation, or flower and fruit development [89]. Various *Bacillus* species have the ability to produce a wide range of different GAs in vitro [90]. In a study by Ji et al. [87], the production of GAs by *B. subtilis* was related to its ability to increase the tolerance of wheat under salinity. Moreover, a study by Kang et al. [91] showed that the production of various GA compounds by *B. tequilensis* was involved in the induction of thermotolerance in soybean plants due to an increase of endogenous jasmonic acid and salicylic acid contents and a downregulation of abscisic acid showing the interaction between the various phytohormones in stress regulation. Abscisic acid (ABA) is considered an essential messenger in the adaptive response of plants to abiotic stress. Under water-deficit conditions, ABA plays a vital role in providing plants the ability to signal to their shoots that they are experiencing stressful conditions around the roots, eventually resulting in water-saving anti-transpirant activity, notably stomatal closure and reduced leaf expansion [92]. ABA upregulates the processes involved in cell turgor maintenance and synthesis of osmoprotectants and antioxidant enzymes conferring desiccation tolerance [93]. Study by Zhang et al. [94] reported a proportional increase in ABA concentration upon exposure of plants to salinity. A study by Pan et al. [95] also reported that the production of ABA by *B. subtilis* resulted in an increased tolerance to the heavy metal cadmium in *Brassica chinesis* plant by reducing cadmium-induced photosynthesis inhibition and the oxidative damage in plant tissues through increased levels of antioxidant compounds.

Although many studies have shown that there is a positive correlation between *Bacillus* synthesized phytohormones and plant growth and development under various abiotic stresses, there is a lack of knowledge of the underlying biochemical mechanisms involved in the enhanced responses to abiotic stresses. Thus, to decipher all the chemical spheres and pathways involved in the interaction between *Bacillus* phytohormones and the plant under various environmental stresses, there is a need for more systemic biology studies. Apart from phytohormones, *Bacillus* spp. also produce VOCs, which induce abiotic stress tolerance. *Bacillus* VOCs have the potential to simultaneously up-regulate high affinity *HKT1* in shoots and down-regulate it in the roots, thus enhancing HKT1-dependent shoot-to-root sodium ion circulation, which is crucial in salinity tolerance. This was observed in a study by Zhang et al. [96] that VOCs such as 2,3-butanediol produced by *B. amyloliquefaciens* GB03 strain reduced accumulation of sodium ions in *Arabidopsis* shoots resulting in enhanced salinity stress tolerance. Zhang et al. [97] reported that exposure of *Arabidopsis* to *Bacillus* VOCs resulted in the accumulation of high levels of choline and glycine betaine, which are vital osmo-protectants that confer drought tolerance in plants [98].

The molecular mechanisms governing *Bacillus*-mediated abiotic stress tolerance are not limited to *Bacillus*’ ability to produce phytohormones and VOCs. Other mechanisms associated with enhancing stress tolerance involve triggering biological and physiological processes such as ROS detoxification mechanisms, osmoprotection, stomatal regulation, membrane stability, xylem hydraulic conductance, root zone water, and nutrient availability and metal chelation [99,100,101]. For example, recent studies by Nephali et al. [11], Lephatsi et al. [13] and Othibeng et al. [102] revealed that the application of *Bacillus* consortium (a commercial biostimulant known as Bacstim^®^ 100) to maize plants improves drought tolerance via mechanism such as enhanced energy production, enhanced expression of drought stress responsive defense genes, osmoregulation, redox homeostasis, strengthening of the plant cell wall and membrane remodeling.

As highlighted herein, there is vast knowledge on the *Bacillus*–plant interactions leading to growth promotion and stress resistance. However, there is still a lack of fundamental research that provides direct mechanisms of action underlying *Bacillus*–plant interactions. Thus, further research on the components and effects of *Bacillus*-based biostimulants on the plant health and growth is required to advance the development of a scientifically based biostimulant industry with the potential to encourage effective exploration and application of *Bacillus*-based biostimulants in agriculture for improved and sustainable food security. Comprehensive study approaches such as “*omics*” sciences have shown great potential to interrogate the metabolism of plants and *Bacillus* to generate mechanistic models with actionable knowledge and insights on how *Bacillus* promotes plant growth and enhances defense responses.

## 5. *Omics* Sciences in Studying *Bacillus* spp. and Its Effects on Plants: Metabolomics Applications

**Figure 4:**
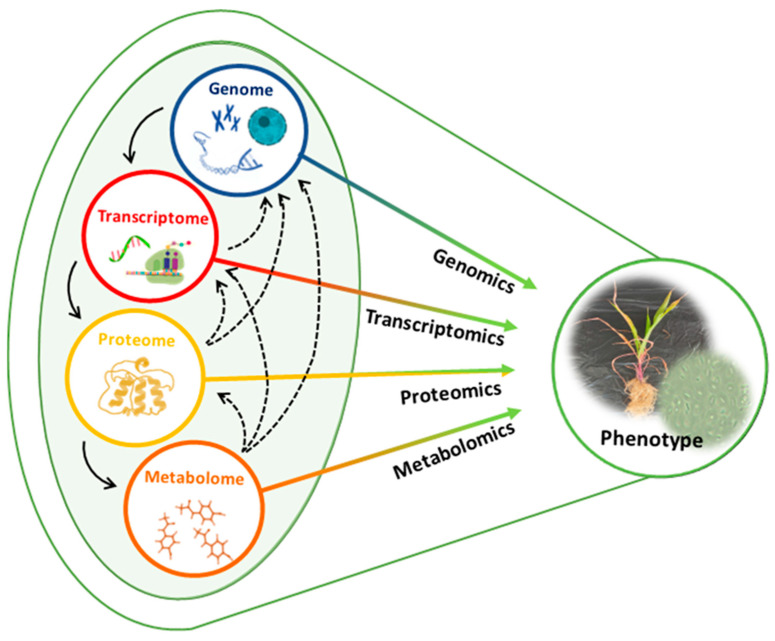
*Omics* technologies in systems biology. The *omics* cascade shows the flow of information from gene level to the metabolome with metabolomics being the closest link to the phenotype.

*Omics* refers to a biological study field where a comprehensive assessment of molecular entities is performed at different levels in the systems biology, such as DNA (genomics), RNA (transcriptomics), proteins (proteomics), and other cellular molecules (metabolomics) ([103]; Figure 4). Advancements in these technologies have proven instrumental in decoding important multilayered biochemical events underpinning the effects of *Bacillus*-based biostimulants on plants [99,104,105,106]. Metabolomics is a multidisciplinary *omics* science which offers a comprehensive identification and quantitation of low-molecular-weight molecules, namely metabolites. Plant metabolites are considered the “cause of plant phenotype” [107]. As such, the application of metabolomics in investigating the rhizospheric complex and dynamic chemical communications between *Bacillus* and plants would be of great value to agricultural research [108].

The belowground intercommunication involves inter-and intra-organismal interactions (i.e., plant–*Bacillus*, plant-to-plant, and *Bacillus*–other microbes) [109]. Such multidimensional interactions are facilitated and controlled through the interconnected belowground signaling networks, resulting in altered plant growth and development. Thus far, as highlighted in Section 3, the known signaling molecules and metabolites include root exudates, volatile organic molecules (VOCs), allelochemicals, lytic enzymes, toxins, and quorum sensing molecules (QSM), phytohormones, siderophores, lipopeptides, and lytic enzymes [110,111]. While some studies have made significant contributions to characterizing the composition of rhizobiomes, there are still considerable gaps in understanding how plants shape their rhizobiomes and how the bacterial presence and exudates affect the plant and its root microbiota [29,112].

Considering the intricate complexities in the rhizosphere mentioned above, studying these interactions concertedly is challenging and almost impractical. Thus, the existing literature mainly focuses on investigating one or two of the interacting partners to intently extract useful and accurate information that can offer the best description of these dynamic interactions. As such, the following sections discuss metabolomics studies aimed at investigating (i) *Bacillus* intracellular and extracellular chemical space, (ii) interactions between *Bacillus* and other microbes in the rhizosphere, and (iii) *Bacillus* and plant intercommunications.

### 5.1. Application of Metabolomics in Investigating Bacillus Chemical Space

The emergence of *omics* sciences such as metabolomics allows a comprehensive examination of biological systems at a global level and the exploration of interrelationships and dynamics between the multi-component systems, i.e., holobiont [113]. The untargeted metabolomics studies interrogating the chemical space of *Bacillus* are still very limited; however, most studies employ analytical platforms such as chromatography coupled to mass spectrometry and nuclear magnetic resonance (NMR) spectroscopy (commonly utilized in metabolomics) to structurally characterize bacterial metabolites (Table 3). For example, the application of LC-MS and NMR spectroscopy revealed the identity of two novel cyclic depsipeptides turnagainolides A and B and that these compounds contain a combination of R and S amino acids and the (E)-3- hydroxy-5-phenylpent-4-enoic acid fragment Hppa, which are hallmarks of nonribosomal peptide biosynthesis [114].

In another study, LC-MS and NMR spectroscopy-guided metabolic profiling was employed and two new cyclic-lipotetrapeptides, bacilotetrins A and B were identified in the extracellular milieu of *B. subtilis* [115]. A cyclic lipopeptide plipastatin A1 was identified from the extracellular milieu *B. amyloliquefaciens* SH-B74 using a combination of analytical platforms including LC-MS, GC-MS, and NMR [119]. The cumulative findings of such studies led to wide-ranging secondary metabolites that are known to be present in various *Bacillus* strains (Table 3). Such metabolites include peptides of low molecular weight that are generated ribosomally (bacteriocins) or non-ribosomally; lipopeptides such as surfactins, iturins, and fengycins and polyketides such as macrolactins, difficidins, and oxidifficidins [116]. Interrogating and profiling the intracellular and extracellular metabolome of the *Bacillus* strains will provide insightful science-based information that can be leveraged in designing and formulating novel *Bacillus* biostimulants. Moreover, understanding the intracellular metabolome of *Bacillus* and the metabolites they secrete to the exterior, can enhance the understanding of *Bacillus*-plant intercommunication [12,133].

With innovative developments in analytical technologies (integrating artificial intelligence, AI and machine learning, ML), advancements in chemometrics and statistical methods (big data analytics and management), and the integration of orthogonal biological approaches, untargeted metabolomics is becoming a fundamental fulcrum to creating a “*Rosetta stone*” for deciphering the encryptions existing in the rhizosphere ecology [134,135]. One of the emerging analytical aspects is ion mobility technologies such as ion mobility spectrometry (IMS). Interfacing IMS with mass spectrometry (IMS-MS) increases the analytical power, enabling the efficient separation, resolution, identification, and multidimensional structural characterization of analytes [136,137]. Currently, there are limited studies demonstrating the application of IMS technologies in investigating the *Bacillus* spp. One of the very few studies include a recent study by Ratiu et al. [138], where a portable aspiration-type ion mobility spectrometer (a-IMS) and gas chromatography-mass spectrometry (GC-MS) were applied to discriminate between different bacteria, *Escherichia coli*, *Bacillus subtilis* and *Staphylococcus aureus*, by rapid sensing of the bacterial metabolic volatiles, which produced differential metabolic fingerprints.

Another analytical tool that has proven instrumental for profiling live microbial colonies is nanoDESI MS [134]. The applications of this technique allowed highly sensitive metabolic profiling directly off living microbial communities, requiring zero sample preparation. Other advantages of this tool include the ability to capture a wide variety of metabolite families within a single mass spectrum directly from a live specimen thus giving comprehensive visuals into the chemical space of bacteria. Such insights are fundamental to describing the microbial chemotype that are more accurate to the phenotype [134]. For example, in this study by Watrous et al. [134], nanoDESI MS and MS/MS molecular networks demonstrated an elevation of metabolite content within *B. subtilis* across the 60 h time growth period, with increased production of structural variants of the cyclic peptides surfactin, plipastatin, and subtilosin steadily increasing over time.

Furthermore, some of the recent advancements in data handling and metabolite annotation include fourth industrial revolution (4IR)-inspired computational tools such as molecular networking (MN) integrated with in silico annotation tools [139,140,141] are positively impacting the chemical and biological interpretation of untargeted metabolomics studies on *Bacillus* and *Bacillus*–plant interactions. The application of MN in microbial metabolomics enabled detection and visualization of related metabolites in *Bacillus* cells via spectral similarities within and between data sets as chemical families are grouped together [12,134]. For instance, the study by Nephali et al. [12] applied MN tools to profile the intracellular chemical space of PGPR *Bacillus* strains: *B. laterosporus*, *B. amyloliquefaciens*, *B. licheniformis* 1001, and *B. licheniformis* M017 and their consortium—this study showed higher content of surfactins in *Bacillus* consortium and *B. amyloliquefaciens* and higher content of lichenysins *B. licheniformis* strains compared to other strains under study. Additionally, MN proved instrumental to uncovering the temporal dynamics in *B. subtilis* (Watrous et al., 2012) and *B. laterosporus*, *B. amyloliquefaciens*, *B. licheniformis* 1001, and *B. licheniformis* M017 [12]. Time-dependent MN showed an increasing number of lipopeptides over time in *B. amyloliquefaciens*, *B. licheniformis* 1001, *B. licheniformis* M017, and *B. laterosporus* and their consortium, and the stationary phase showed the highest content of lipopeptides in the cells [12]. A recent study by Wang et al. [142] revealed novel compounds Hetiamacin E and F from *B. subtilis* PJS using MS/MS-based MN. Another study by Purves et al. [143] demonstrated the application of metabolomics and MN for microbial secondary metabolite bioprospecting. Decoding metabolic profiles of *Bacillus* strains will contributes toward understanding the chemical space of PGPR, which will subsequently help in decoding the chemical communication underlying PGPR-plant interactions.

Despite the increasing advancements in the metabolomics workflow mentioned above, the chemical space of *Bacillus* is still not fully elucidated. Currently, the metabolites that have been found in *Bacillus* include amino acids, hormones, organic acids, nucleotides, cofactors, and sugar-phosphates [144] and structurally diverse secondary metabolites, such as lipopeptides (e.g., iturins, fengycins, and surfactins), polyketides, polypeptides, macrolactones, lipoamides, fatty acids, isocoumarins [53,145,146], siderophores such as hydroxamates (e.g., schizokinen, pyochelin), catecholates (e.g., bacillibactin, petrobactin), and carboxylates (e.g., rhizobactin) [147,148]. Identification of these metabolites in *Bacillus* cells have contributed toward understanding the biochemistry of *Bacillus* and their interaction with the host plants and other microorganism. For example, metabolites such as lipopeptides, macrolides, and polyketide have been demonstrated to possess antimicrobial and antifungal activities (Table 2), mechanisms that are involved in biocontrol of phytopathogens [148]. Moreover, other bacterial metabolites such as hormones (indole acetic acid) and amino acids (tryptophan) have been shown to promote plant growth [148]. However, the current knowledge is barely a tip of the iceberg, as a large percentage of *Bacillus* metabolome remains unexplored [144,149]. Comprehensively annotating and characterizing the metabolome of *Bacillus* will advance our understanding of belowground chemical communications between *Bacillus* and plants. Such accurate models would explain mechanisms of action of *Bacillus*, molecular events that govern *Bacillus*-mediated enhanced plant growth and stress protection. This actionable knowledge is necessary for innovatively designing and implementing *Bacillus*-based formulations for sustainable agriculture.

### 5.2. Application of Metabolomics in Investigating Bacillus and Other Microorganisms’ Interactions in the Rhizosphere

The holobiont dynamics (assembly of the phytomicrobiome) involves the chemical intercommunications between the microbe–microbe interactions and plant–microbe interactions [150]. As rhizosphere-dwelling bacteria, *Bacillus* are surrounded and constantly interacting with other microbes via different types of communications [151]. The intercellular interactions between microbes occur via four main mechanisms such as cell–cell signaling, production of secondary metabolites, cell–cell contacts and metabolic interplay [152]. The cell–cell signaling involves the interexchange of diverse chemical signaling molecules such as volatiles, quorum sensing signals, and secondary metabolites to communicate, regulate, and synchronise microbial behaviors. However, this signal network is not well understood—there are knowledge gaps in determining the predominance, diversity, and function of signaling molecules and how such factors drive the communal behavior and population dynamics within phytomicrobiome ecosystem.

As such, metabolomics approaches can be employed to bridge the existing knowledge gaps [108,150,153]. One of the very few is a study by Wen et al. [154], which demonstrated that several bacterial groups such as *Bacillus* and *Chitinophaga* were negatively related to the pathogen abundance. The GC-MS analyses revealed significantly different metabolomes in two groups of rhizosphere soils, i.e., the rhizosphere soil of lower harbored more sugars such as fructose, sucrose and melibiose than that in high pathogen abundance, indicated their potential biocontrol ability. A recent study by Andric et al. [155] applied a highly advanced analytical platform, MALDI-FT-ICR MS imaging, revealing that *Bacillus* mobilizes its cyclic lipopeptide surfactin to improve motility and reduce the toxicity of *Pseudomonas* by acting as a chemical deactivator of *Pseudomonas* lipopeptides, sessilins and tolaasins.

The secretion of lipopeptides surfactins by *Bacillus* displays an antagonist relationship with other microbes and has also been shown to exert a symbiotic relationship with other bacterial species, thus shaping the plant microbiome. For example, the study by Luzzatto-Knaan et al. [133], demonstrated the role of surfactins as an interspecies recruitment factor. In this study by Luzzatto-Knaan et al. [133], mass spectrometry and MN approaches were applied and revealed that *B. subtilis* secretes surfactins to recruit *Paenibacillus dendritiformis* to its ecological niche and that *P. dendritiformis* actively degrades surfactins originating from *B. subtilis* and marks its territory by accumulating the resulting surfactin degradation products. Such studies illustrate the indispensability of metabolomics in revealing the ecological roles of secondary metabolites such as lipopeptides during microbial interspecies interactions as well as the regulation of their expression under naturally competitive soil conditions [133,155]. Moreover, studies like these bring the scientific community much closer to fully understanding the mechanisms of *Bacillus* mechanisms for agricultural applications.

### 5.3. Application of Metabolomics in Investigating the Bacillus and Plant Intercommunications

Various metabolomics studies have illustrated that the *Bacillus*–plant interaction (which may involve the secretion of secondary metabolites from *Bacillus*, Table 2) can confer enhanced growth-promotion and defense priming of the plant through the reprogramming of the plant metabolome [146]. For example, a study by Nephali et al. [11] revealed an increased pool of tricarboxylic acid (TCA) intermediates, reprogramming of amino acid profiles and differential changes in phenolics and lipids as key metabolomics signatures induced by the application of *Bacillus*-based biostimulants on maize plants. Another metabolomics by Kang et al. [156], showed decreased levels of glucose, fructose, sucrose, and trehalose in *B. simplex*-treated soybean roots compared to the control group—decreased sugar levels was translated to reduced food sources for nematodes. Furthermore, treatment with *B. simplex* led to higher levels of melibiose, gluconic acid, lactic acid, phytosphingosine, and noradrenaline in soybean roots, which promoted nematocidal activity thus, improving disease resistance.

A metabolomics approach was also applied to reveal the underlying mechanisms employed by *Pa. alvei* NAS-6G6 and *B. velezensis* N54 in plant protection against biotic (*Fusarium pseudograminearum* crown rot) and abiotic (drought) stress. In this study, *Pa. alvei* NAS-6G6 was found to induce unique protection capacity against biotic and abiotic stress, and combined stresses by upregulating different defense metabolites *S. bicolor* plants and altering metabolic pathways such as riboflavin metabolism under biotic and drought stress and glutathione metabolism under combined biotic (*Fusarium pseudograminearum* crown rot) and abiotic (drought) stress. *B. velezensis* N54 upregulated arginine and proline metabolism, *Pa. alvei* NAS-6G6 upregulated riboflavin metabolism under biotic stress condition (*Fusarium pseudograminearum* crown rot) [157]. Riboflavin and proline are known to plays significant roles in regulating antioxidant mechanisms and osmoprotection [157]. A recent metabolomics study by Shahid et al. [146], profiled extracellular secondary metabolites and hormones from *Bacillus* spp., which positively correlated to plant growth promotion and antifungal properties. The application of metabolomics to investigate the effects of *Bacillus* and *Bacillus*-based formulations is gaining some momentum, thus generating a wealth of knowledge driving toward the complete elucidation of *Bacillus*-mediated plant growth promotion and stress protection mechanisms. Thus, a reader is referred to review papers by Nephali et al. [99] and Lephatsi et al. [13] for more details on applying metabolomics approaches in studying microbe-plant–stress interactions.

*Bacillus* spp. are emerging as key microorganisms in the biostimulant industry for maintaining sustainable food security. However, as reviewed in this manuscript there are still major knowledge gaps and bottlenecks in the formulation of *Bacillus*-based biostimulants. This includes poor characterization of active components and synergies in the biostimulant composition and undefined mechanisms and modes of action of biostimulant products, at cellular and molecular levels. Moreover, it is difficult to predict the field application efficacy of these biostimulants due to various environmental factors that may affect field crops, such the soil chemistry, biotic and abiotic stresses. These limitations hinder not only the designing of novel formulations and biostimulant-based agricultural strategies but also the establishment of a standardized legislative framework and regulatory system for the biostimulant industry. Metabolomics, a multidisciplinary *omics* science, offers unique opportunities to predictively decode the mechanisms and modes of action of biostimulants on crop plants, and elucidating signatory markers and metabolic profiles that define the biostimulant action (Table 2). The application of metabolomics in biostimulant research can help us to comprehensively decipher the molecular basis behind biostimulant-induced plant metabolomic reprograming which leads to improved plant health, growth, and increased yield. Such fundamental and indispensable knowledgebase would improve our understanding of biostimulants, also providing a roadmap for translational applications—designing of novel formulations and devising biostimulant-based precision agricultural programs and modules—for sustainable food security.

## 6. Conclusions and Future Prospects

In this critical review we narratively revisited the importance of PGPR such as those belonging to the genus *Bacillus* in improving plant growth and enhancing plant protection against adverse environmental factors. Furthermore, the incorporation of biostimulants, such as microbial formulations (e.g., *Bacillus*-based products), in cropping systems has increasingly shown to be a promising strategy for sustainable agriculture and global food security, aligning with the United Nations sustainable development goals (SDGs). Despite the current wealth of knowledge on *Bacillus* spp. and its applications, molecular mechanisms that govern modes of action of *Bacillus*-based formulations remain poorly understood. Thus, this review suggests that *omics* sciences, with a focus on metabolomics, offer unique opportunities to illuminate the chemical intercommunications between *Bacillus* and plants, and to elucidate biochemical and molecular details on modes of action of *Bacillus*-based formulations.

## Figures and Tables

**Table 1:** *Bacillus* spp. biostimulant formulations.

Microorganism	Formulation Type	Additives	References
Consortium * of *Bacillus licheniformis*, *Bacillus laterosporus* and *Bacillus amyloliquefaciens*	Liquid	No additives disclosed	[11,12,13]
*Bacillus megaterium*	Granules	Lactose monohydrate PVPK-30, sodium alignate	[14]
*Bacillus megaterium*	Powder	Talc, clay and cellulose; CMC, sodium benzoate, CaCO_3_, Glucose, sucrose, mannitol, yeast, peptone.	[15]
*Bacillus amyloliquefacines*	Powder	Sucrose, powder skimmed milk, MgSO_4_	[16]
*Bacillus amyloliquefacines*	Liquid	Sucrose, powder skimmed milk, MgSO_4_	[16]
*Bacillus amyloliquefacines*	Powder	MgSO_4_	[17]
*Bacillus cereus*	Powder	Glucose, fructose, D-galactose, sucrose, trehalose, cellobiose, glutamic acid, soluble starch, glycerol, sorbitol, peptone, nonfat skimmed milk.	[18]
*Bacillus cereus*	Powder	Talc, CMC, CaCO_3,_ Glucose	[19]
*Bacillus subtilis*	Powder	Soybean flour	[20]
*Bacillus subtilis*	Liquid	Groundnut oil, Pongamia oil and sunflower oil; glycerol	[21]
*Bacillus subtilis* and *licheniformis*	Powder	Natural zeolite Synthetic zeolite	[21]

* This consortium is on the market as a biostimulant product, BACSTIM^®^ 100.

**Table 2:** Effects of VOCs produced by *Bacillus*.

VOCs	*Bacillus* spp.	Plant	Response	References
2,3-Butandiol	*B. subtilis*; *B. amyloliquenfaciens*	*Arabidopsis thaliana*, *Solanum tuberusom*	Induced systemic resistance/tolerance, plant growth promotion	[47,48]
Butyrolactone	*B. cereus*, *B. subtilis*	*Arabidopsis thaliana*	Growth promotion, modify root system architecture	[49]
Acetophenone	*B. megaterium*, *B. pumilis*	*Arabidopsis thaliana*	Growth promotion, modify root system architecture	[49]
2-Butanone	*B. subtilis*; *B. amyloliquenfaciens*	*Nicotinia tabacum*	Growth promotion	[50]
3-Pentanol	*B. megaterium*, *B. pumilis*	*Capsicum annum*	Induced systemic resistance, plant growth promotion	[51]

**Table 3:** Identified metabolites in the intracellular and extracellular chemical space of *Bacillus* spp.

*Bacillus* Species	Metabolite	Chemical Nature	Bioactivity	References
*B. subtilis* (unidentified marin starin)	Bacilotetrins	Cyclic-lipotetrapeptides	Antimicrobial	[115]
*B. amyloliquefaciens* AP183	Bacillusin	Macrocyclic polyene	Antimicrobial	[116]
*B. subtilis* (unidentified marin starin)	Gageotetrins	Linear lipopeptides	Antimicrobial, anticancer	[117]
*B. subtilis* DSM 16696	Macrolactin	Macrolides	Antimicrobial	[118]
*B. subtilis* DSM 16697	Plipastatin A	Lipopeptides	Antifungal	[119]
*B. subtilis* MTCC 10403	Furanoterpenoids	Polyketide	Antimicrobial	[120]
Unidentified *Bacillus* strain	Turnagainolides	Depsipeptides	Activation of SHIP1	[114]
*B. subterraneus* 11593	Bacilsubteramide A	Alkaloid		[121]
*B. cereus* RKHC-09	Cereusitin A	Cyclic tetrapeptide	Antifungal	[122]
*B. amyloliquefaciens* HAB-2	Bacillomycin	Cyclic lipopeptide	Antifungal	[123]
*Bacillus* sp. FS8D	Pseurotin A	Spirocyclic	Anticancer	[124]
*B. coagulans* 14	Coagulin	Peptide	Antibacterial	[125]
*B. thuringenesis*	Bacthurucin f4	Peptide	Antifungal	[126]
*B. cereus*	Cerein	Peptide	Antibacterial	[127]
*B. megaterium*	Megacin	Peptide	Antibacterial	[128]
*B. thuringenesis* S	Thuricin	Peptide		[129]
*B. licheniformis*	Halobacillin 5b	Hemolytic, cytotoxic		[130]
*B. amyloliquefaciens* GSB272	Bacilysin 1	Antifungal, antibacterial		[131]
*B. subtilis* 168	Bacilysocin	Fungicidal, antibacterial		[132]
*B. licheniformis* 1001, *B. licheniformis* M017, *B. amyloliquefaciens*	Lichenysins Surfactins	antimicrobials		[12]

## Data Availability

Not applicable.

## References

[1] Kleinwechter U., Gastelo M., Ritchie J., Nelson G., Asseng S. (2016). Simulating cultivar variations in potato yields for contrasting environments. Agric. Syst..

[2] Radhakrishnan R., Hashem A., Abd Allah E.F. (2017). *Bacillus*: A biological tool for crop improvement through bio-molecular changes in adverse environments. Front. Physiol..

[3] Bawa A.S., Anilakumar K.R. (2013). Genetically modified foods: Safety, risks and public concerns—A review. J. Food Sci. Technol..

[4] Alengebawy A., Abdelkhalek S.T., Qureshi S.R., Wang M.Q. (2021). Heavy metals and pesticides toxicity in agricultural soil and plants: Ecological risks and human health implications. Toxics.

[5] Backer R., Rokem J.S., Ilangumaran G., Lamont J., Praslickova D., Ricci E., Subramanian S., Smith D.L. (2018). Plant Growth-Promoting Rhizobacteria: Context, Mechanisms of Action, and Roadmap to Commercialization of Biostimulants for Sustainable Agriculture. Front. Plant Sci..

[6] Grover M., Ali S.Z., Sandhya V., Rasul A., Venkateswarlu B. (2011). Role of microorganisms in adaptation of agriculture crops to abiotic stresses. World J. Microbiol. Biotechnol..

[7] Vejan P., Abdullah R., Khadiran T., Ismail S., Nasrulhaq Boyce A. (2016). Role of plant growth promoting rhizobacteria in agricultural sustainability—A review. Molecules.

[8] Siddikee M.A., Chauhan P.S., Anandham R., Han G.H., Sa T. (2010). Isolation, characterization, and use for plant growth promotion under salt stress, of ACC deaminase-producing halotolerant bacteria derived from coastal soil. J. Microbiol. Biotechnol..

[9] Beauregard P.B., Chai Y., Vlamakis H., Losick R., Kolter R. (2013). *Bacillus subtilis* biofilm induction by plant polysaccharides. Proc. Natl. Acad. Sci. USA.

[10] Shafi J., Tian H., Ji M. (2017). *Bacillus* species as versatile weapons for plant pathogens: A review. Biotechnol. Biotechnol. Equip..

[11] Nephali L., Moodley V., Piater L., Steenkamp P., Buthelezi N., Dubery I., Burgess K., Huyser J., Tugizimana F. (2021). A Metabolomic Landscape of Maize Plants Treated with a Microbial Biostimulant under Well-Watered and Drought Conditions. Front. Plant Sci..

[12] Nephali L., Steenkamp P., Burgess K., Huyser J., Brand M., van der Hooft J.J.J., Tugizimana F. (2022). Mass Spectral Molecular Networking to Profile the Metabolome of Biostimulant *Bacillus* Strains. Front. Plant Sci..

[13] Lephatsi M., Nephali L., Meyer V., Piater L.A., Buthelezi N., Dubery I.A., Opperman H., Brand M., Huyser J., Tugizimana F. (2022). Molecular mechanisms associated with microbial biostimulant-mediated growth enhancement, priming and drought stress tolerance in maize plants. Sci. Rep..

[14] Chumthong A., Kanjanamaneesathian M., Pengnoo A., Wiwattanapatapee R. (2008). Water-soluble granules containing *Bacillus* megaterium for biological control of rice sheath blight: Formulation, bacterial viability and efficacy testing. World J. Microbiol. Biotechnol..

[15] Omer A.M. (2010). Bioformulations of bacillus spores for using as Biofertilizer. Life Sci. J..

[16] Gotor-Vila A., Usall J., Torres R., Abadias M., Teixidó N. (2017). Formulation of the biocontrol agent *Bacillus amyloliquefaciens* CPA-8 using different approaches: Liquid, freeze-drying and fluid-bed spray-drying. BioControl.

[17] Han L., Pu T., Wang X., Liu B., Wang Y., Feng J., Zhang X. (2018). Optimization of a protective medium for enhancing the viability of freeze-dried *Bacillus amyloliquefaciens* B1408 based on response surface methodology. Cryobiology.

[18] Martínez-Álvarez J.C., Castro-Martínez C., Sánchez-Peña P., Gutiérrez-Dorado R., Maldonado-Mendoza I.E. (2016). Development of a powder formulation based on *Bacillus cereus* sensu lato strain B25 spores for biological control of Fusarium verticillioides in maize plants. World J. Microbiol. Biotechnol..

[19] Mahidsanan T., Gasaluck P., Eumkeb G. (2017). A novel soybean flour as a cryoprotectant in freeze-dried *Bacillus subtilis* SB-MYP-1. LWT Food Sci. Technol..

[20] Jayasudha S.M., Kirankumar K.C., Mesta R.K., Ippikoppa R. (2018). Liquid Formulation Using Different Oils and Shelf Life Study of Effective Bacterial Bio-Agents. Int. J. Curr. Microbiol. Appl. Sci..

[21] Chung S., Lim J.H., Kim S.D. (2010). Powder formulation using heat resistant endospores of two multi-functional plant growth promoting rhizobacteria *Bacillus* strains having phytophtora blight suppression and growth promoting functions. J. Appl. Biol. Chem..

[22] Haas D., Défago G. (2005). Biological control of soil-borne pathogens by fluorescent pseudomonads. Nat. Rev. Microbiol..

[23] Wilson B.R., Bogdan A.R., Miyazawa M., Hashimoto K., Tsuji Y. (2016). Siderophores in Iron Metabolism: From Mechanism to Therapy Potential. Trends Mol. Med..

[24] Sabir S., Arshad M., Chaudhari S.K. (2014). Zinc oxide nanoparticles for revolutionizing agriculture: Synthesis and applications. Sci. World J..

[25] Arkhipova T.N., Veselov S.U., Melentiev A.I., Martynenko E.V., Kudoyarova G.R. (2005). Ability of bacterium *Bacillus subtilis* to produce cytokinins and to influence the growth and endogenous hormone content of lettuce plants. Plant Soil.

[26] Ryu C.M., Faragt M.A., Hu C.H., Reddy M.S., Wei H.X., Paré P.W., Kloepper J.W. (2003). Bacterial volatiles promote growth in Arabidopsis. Proc. Natl. Acad. Sci. USA.

[27] Zhang H., Kim M.S., Krishnamachari V., Payton P., Sun Y., Grimson M., Farag M.A., Ryu C.M., Allen R., Melo I.S. (2007). Rhizobacterial volatile emissions regulate auxin homeostasis and cell expansion in Arabidopsis. Planta.

[28] Xie S.S., Wu H.J., Zang H.Y., Wu L.M., Zhu Q.Q., Gao X.W. (2014). Plant growth promotion by spermidine-producing *Bacillus subtilis* OKB105. Mol. Plant-Microbe Interact..

[29] Sasse J., Martinoia E., Northen T. (2018). Feed Your Friends: Do Plant Exudates Shape the Root Microbiome?. Trends Plant Sci..

[30] Shaikh S.S. (2018). Impact of Interactions between Rhizosphere and Rhizobacteria: A Review Plant Microbes interaction View project Soil Bioremediation: An approach towards sustainable cleaner technology View project. J. Bacteriol. Mycol..

[31] Odoh C.K. (2017). Plant Growth Promoting Rhizobacteria (PGPR): A Bioprotectant bioinoculant for Sustainable Agrobiology. A Review. Int. J. Adv. Res. Biol. Sci..

[32] Poveda J., González-Andrés F. (2021). *Bacillus* as a source of phytohormones for use in agriculture. Appl. Microbiol. Biotechnol..

[33] Shao J., Li S., Zhang N., Cui X., Zhou X., Zhang G., Shen Q., Zhang R. (2015). Analysis and cloning of the synthetic pathway of the phytohormone indole-3-acetic acid in the plant-beneficial *Bacillus amyloliquefaciens* SQR9. Microb. Cell Fact..

[34] Shameer S., Prasad T.N.V.K.V. (2018). Plant growth promoting rhizobacteria for sustainable agricultural practices with special reference to biotic and abiotic stresses. Plant Growth Regul..

[35] Wagi S., Ahmed A. (2019). *Bacillus* spp.: Potent microfactories of bacterial IAA. PeerJ.

[36] Karadeniz A., Topcuoǧlu Ş.F., Inan S. (2006). Auxin, gibberellin, cytokinin and abscisic acid production in some bacteria. World J. Microbiol. Biotechnol..

[37] Kudoyarova G.R., Korobova A.V., Akhiyarova G.R., Arkhipova T.N., Zaytsev D.Y., Prinsen E., Egutkin N.L., Medvedev S.S., Veselov S.Y. (2014). Accumulation of cytokinins in roots and their export to the shoots of durum wheat plants treated with the protonophore carbonyl cyanide m-chlorophenylhydrazone (CCCP). J. Exp. Bot..

[38] Rizza A., Jones A.M. (2019). The makings of a gradient: Spatiotemporal distribution of gibberellins in plant development. Curr. Opin. Plant Biol..

[39] Radhakrishnan R., Lee I.J. (2016). Gibberellins producing *Bacillus methylotrophicus* KE2 supports plant growth and enhances nutritional metabolites and food values of lettuce. Plant Physiol. Biochem..

[40] Tyagi S., Mulla S.I., Lee K.J., Chae J.C., Shukla P. (2018). VOCs-mediated hormonal signaling and crosstalk with plant growth promoting microbes. Crit. Rev. Biotechnol..

[41] Lemfack M.C., Nickel J., Dunkel M., Preissner R., Piechulla B. (2014). MVOC: A database of microbial volatiles. Nucleic Acids Res..

[42] Park Y.S., Dutta S., Ann M., Raaijmakers J.M., Park K. (2015). Promotion of plant growth by Pseudomonas fluorescens strain SS101 via novel volatile organic compounds. Biochem. Biophys. Res. Commun..

[43] Kanchiswamy C.N., Malnoy M., Maffei M.E. (2015). Chemical diversity of microbial volatiles and their potential for plant growth and productivity. Front. Plant Sci..

[44] Xu Y.Y., Lu H., Wang X., Zhang K.Q., Li G.H. (2015). Effect of Volatile Organic Compounds from Bacteria on Nematodes. Chem. Biodivers..

[45] Kai M. (2020). Diversity and Distribution of Volatile Secondary Metabolites Throughout *Bacillus subtilis* Isolates. Front. Microbiol..

[46] Piechulla B., Lemfack M.C., Kai M. (2017). Effects of discrete bioactive microbial volatiles on plants and fungi. Plant Cell Environ..

[47] Rybakova D., Rack-Wetzlinger U., Cernava T., Schaefer A., Schmuck M., Berg G. (2017). Aerial warfare: A volatile dialogue between the plant pathogen Verticillium longisporum and its antagonist Paenibacillus polymyxa. Front. Plant Sci..

[48] Farag M.A., Ryu C.M., Sumner L.W., Paré P.W. (2006). GC-MS SPME profiling of rhizobacterial volatiles reveals prospective inducers of growth promotion and induced systemic resistance in plants. Phytochemistry.

[49] Tahir H.A.S., Gu Q., Wu H., Raza W., Hanif A., Wu L., Colman M.V., Gao X. (2017). Plant growth promotion by volatile organic compounds produced by *Bacillus subtilis* SYST2. Front. Microbiol..

[50] Fincheira P., Venthur H., Mutis A., Parada M., Quiroz A. (2016). Growth promotion of Lactuca sativa in response to volatile organic compounds emitted from diverse bacterial species. Microbiol. Res..

[51] Choi H.K., Song G.C., Yi H.S., Ryu C.M. (2014). Field Evaluation of the Bacterial Volatile Derivative 3-Pentanol in Priming for Induced Resistance in Pepper. J. Chem. Ecol..

[52] Prithiviraj B., Perry L.G., Badri D.V., Vivanco J.M. (2007). Chemical facilitation and induced pathogen resistance mediated by a root-secreted phytotoxin. New Phytol..

[53] Carvalhais L.C., Muzzi F., Tan C.H., Hsien-Choo J., Schenk P.M. (2013). Plant growth in Arabidopsis is assisted by compost soil-derived microbial communities. Front. Plant Sci..

[54] Chagas F.O., Pessotti R.D.C., Caraballo-Rodríguez A.M., Pupo M.T. (2018). Chemical signaling involved in plant-microbe interactions. Chem. Soc. Rev..

[55] Bi S., Sourjik V. (2018). Stimulus sensing and signal processing in bacterial chemotaxis. Curr. Opin. Microbiol..

[56] Ankati S., Podile A.R. (2019). Metabolites in the root exudates of groundnut change during interaction with plant growth promoting rhizobacteria in a strain-specific manner. J. Plant Physiol..

[57] Feng H., Zhang N., Fu R., Liu Y., Krell T., Du W., Shao J., Shen Q., Zhang R. (2018). Recognition of dominant attractants by key chemoreceptors mediates recruitment of plant growth-promoting rhizobacteria. Environ. Microbiol..

[58] Yuan J., Zhang N., Huang Q., Raza W., Li R., Vivanco J.M., Shen Q. (2015). Organic acids from root exudates of banana help root colonization of PGPR strain *Bacillus amyloliquefaciens* NJN-6. Sci. Rep..

[59] Cesari A., Paulucci N., López-Gómez M., Hidalgo-Castellanos J., Plá C.L., Dardanelli M.S. (2019). Restrictive water condition modifies the root exudates composition during peanut-PGPR interaction and conditions early events, reversing the negative effects on plant growth. Plant Physiol. Biochem..

[60] Xie X., He Z., Chen N., Tang Z., Wang Q., Cai Y. (2019). The Roles of Environmental Factors in Regulation of Oxidative Stress in Plant. Biomed. Res. Int..

[61] Hashem A., Tabassum B., Fathi Abd_Allah E. (2019). *Bacillus subtilis*: A plant-growth promoting rhizobacterium that also impacts biotic stress. Saudi J. Biol. Sci..

[62] Cazorla F.M., Romero D., Pérez-García A., Lugtenberg B.J.J., De Vicente A., Bloemberg G. (2007). Isolation and characterization of antagonistic *Bacillus subtilis* strains from the avocado rhizoplane displaying biocontrol activity. J. Appl. Microbiol..

[63] Wang T., Liang Y., Wu M., Chen Z., Lin J., Yang L. (2015). Natural products from *Bacillus subtilis* with antimicrobial properties. Chin. J. Chem. Eng..

[64] Kinsinger R.F., Shirk M.C., Fall R. (2003). Rapid surface motility in *Bacillus subtilis* is dependent on extracellular surfactin and potassium ion. J. Bacteriol..

[65] Yasmin S., Zaka A., Imran A., Zahid M.A., Yousaf S., Rasul G., Arif M., Mirza M.S. (2016). Plant growth promotion and suppression of bacterial leaf blight in rice by inoculated bacteria. PLoS ONE.

[66] Zebelo S., Song Y., Kloepper J.W., Fadamiro H. (2016). Rhizobacteria activates (+)-δ-cadinene synthase genes and induces systemic resistance in cotton against beet armyworm (*Spodoptera exigua*). Plant Cell Environ..

[67] Pršić J., Ongena M. (2020). Elicitors of Plant Immunity Triggered by Beneficial Bacteria. Front. Plant Sci..

[68] Pieterse C.M.J., Zamioudis C., Berendsen R.L., Weller D.M., Van Wees S.C.M., Bakker P.A.H.M. (2014). Induced systemic resistance by beneficial microbes. Annu. Rev. Phytopathol..

[69] Geudens N., Martins J.C. (2018). Cyclic lipodepsipeptides from *Pseudomonas* spp.—Biological Swiss-Army knives. Front. Microbiol..

[70] Chowdhury S.P., Uhl J., Grosch R., Alquéres S., Pittroff S., Dietel K., Schmitt-Kopplin P., Borriss R., Hartmann A. (2015). Cyclic lipopeptides of *Bacillus amyloliquefaciens* subsp. plantarum colonizing the lettuce rhizosphere enhance plant defense responses toward the bottom rot pathogen Rhizoctonia solani. Mol. Plant-Microbe Interact..

[71] Ongena M., Jourdan E., Adam A., Paquot M., Brans A., Joris B., Arpigny J.L., Thonart P. (2007). Surfactin and fengycin lipopeptides of *Bacillus subtilis* as elicitors of induced systemic resistance in plants. Environ. Microbiol..

[72] García-Gutiérrez L., Zeriouh H., Romero D., Cubero J., de Vicente A., Pérez-García A. (2013). The antagonistic strain *Bacillus subtilis* UMAF6639 also confers protection to melon plants against cucurbit powdery mildew by activation of jasmonate- and salicylic acid-dependent defence responses. Microb. Biotechnol..

[73] Cawoy H., Mariutto M., Henry G., Fisher C., Vasilyeva N., Thonart P., Dommes J., Ongena M. (2014). Plant defense stimulation by natural isolates of *Bacillus* depends on efficient surfactin production. Mol. Plant-Microbe Interact..

[74] Rodríguez J., Tonelli M.L., Figueredo M.S., Ibáñez F., Fabra A. (2018). The lipopeptide surfactin triggers induced systemic resistance and priming state responses in *Arachis hypogaea* L.. Eur. J. Plant Pathol..

[75] Park K., Park Y.S., Ahamed J., Dutta S., Ryu H., Lee S.H., Balaraju K., Manir M., Moon S.S. (2016). Elicitation of induced systemic resistance of chili pepper by iturin a analogs derived from *Bacillus* vallismortis EXTN-1. Can. J. Plant Sci..

[76] Yang T., Rao Z., Zhang X., Xu M., Xu Z., Yang S.T. (2013). Improved Production of 2,3-Butanediol in *Bacillus amyloliquefaciens* by Over-Expression of Glyceraldehyde-3-Phosphate Dehydrogenase and 2,3-butanediol Dehydrogenase. PLoS ONE.

[77] Rudrappa T., Biedrzycki M.L., Kunjeti G., Donofrio N.M., Czymmek K.J. (2010). The rhizobacterial elicitor acetoin induces systemic resistance in *Arabidopsis thaliana*. Commun. Integr. Biol..

[78] Kierul K., Voigt B., Albrecht D., Chen X.H., Carvalhais L.C., Borriss R. (2015). Influence of root exudates on the extracellular proteome of the plant growth-promoting bacterium *Bacillus amyloliquefaciens* FZB42. Microbiololy.

[79] Tiwari S., Prasad V., Chauhan P.S., Lata C. (2017). *Bacillus amyloliquefaciens* confers tolerance to various abiotic stresses and modulates plant response to phytohormones through osmoprotection and gene expression regulation in rice. Front. Plant Sci..

[80] Meena K.K., Sorty A.M., Bitla U.M., Choudhary K., Gupta P., Pareek A., Singh D.P., Prabha R., Sahu P.K., Gupta V.K. (2017). Abiotic stress responses and microbe-mediated mitigation in plants: The omics strategies. Front. Plant Sci..

[81] Santoyo G., del Orozco-Mosqueda M.C., Govindappa M. (2012). Mechanisms of biocontrol and plant growth-promoting activity in soil bacterial species of *Bacillus* and Pseudomonas: A review. Biocontrol Sci. Technol..

[82] Egamberdieva D., Wirth S.J., Alqarawi A.A., Abd-Allah E.F., Hashem A. (2017). Phytohormones and beneficial microbes: Essential components for plants to balance stress and fitness. Front. Microbiol..

[83] Sorokan A., Veselova S., Benkovskaya G., Maksimov I. (2021). Endophytic strain *Bacillus subtilis* 26D increases levels of phytohormones and repairs growth of potato plants after colorado potato beetle damage. Plants.

[84] Hussain A., Hasnain S. (2009). Cytokinin production by some bacteria: Its impact on cell division in cucumber cotyledons. Afr. J. Microbiol. Res..

[85] Arkhipova T.N., Prinsen E., Veselov S.U., Martinenko E.V., Melentiev A.I., Kudoyarova G.R. (2007). Cytokinin producing bacteria enhance plant growth in drying soil. Plant Soil.

[86] Liu F., Xing S., Ma H., Du Z., Ma B. (2013). Cytokinin-producing, plant growth-promoting rhizobacteria that confer resistance to drought stress in *Platycladus orientalis* container seedlings. Appl. Microbiol. Biotechnol..

[87] Ji C., Wang X., Tian H., Hao L., Wang C., Zhou Y., Xu R., Song X., Liu Y., Du J. (2020). Effects of *Bacillus methylotrophicus* M4-1 on physiological and biochemical traits of wheat under salinity stress. J. Appl. Microbiol..

[88] Sun Z., Liu K., Zhang J., Zhang Y., Xu K., Yu D., Wang J., Hu L., Chen L., Li C. (2017). IAA producing *Bacillus altitudinis* alleviates iron stress in *Triticum aestivum* L. seedling by both bioleaching of iron and up-regulation of genes encoding ferritins. Plant Soil.

[89] Fahad S., Hussain S., Bano A., Saud S., Hassan S., Shan D., Khan F.A., Khan F., Chen Y., Wu C. (2015). Potential role of phytohormones and plant growth-promoting rhizobacteria in abiotic stresses: Consequences for changing environment. Environ. Sci. Pollut. Res..

[90] Pandya N.D., Desai P.V. (2013). Gibberellic acid production by *Bacillus cereus* isolated from the rhizosphere of sugarcane. J. Pure Appl. Microbiol..

[91] Kang S.M., Khan A.L., Waqas M., Asaf S., Lee K.E., Park Y.G., Kim A.Y., Khan M.A., You Y.H., Lee I.J. (2019). Integrated phytohormone production by the plant growth-promoting rhizobacterium *Bacillus tequilensis* SSB07 induced thermotolerance in soybean. J. Plant Interact..

[92] Wilkinson S., Kudoyarova G.R., Veselov D.S., Arkhipova T.N., Davies W.J. (2012). Plant hormone interactions: Innovative targets for crop breeding and management. J. Exp. Bot..

[93] Chaves M.M., Maroco J.P., Pereira J.S. (2003). Understanding plant responses to drought—From genes to the whole plant. Funct. Plant Biol..

[94] Zhang J., Jia W., Yang J., Ismail A.M. (2006). Role of ABA in integrating plant responses to drought and salt stresses. Field Crop. Res..

[95] Pan W., Lu Q., Xu Q.R., Zhang R.R., Li H.Y., Yang Y.H., Liu H.J., Du S.T. (2019). Abscisic acid-generating bacteria can reduce Cd concentration in pakchoi grown in Cd-contaminated soil. Ecotoxicol. Environ. Saf..

[96] Zhang H., Xie X., Kim M.S., Kornyeyev D.A., Holaday S., Paré P.W. (2008). Soil bacteria augment Arabidopsis photosynthesis by decreasing glucose sensing and abscisic acid levels in planta. Plant J..

[97] Zhang H., Murzello C., Sun Y., Kim M.S., Xie X., Jeter R.M., Zak J.C., Dowd S.E., Paré P.W. (2010). Choline and osmotic-stress tolerance induced in arabidopsis by the soil microbe *Bacillus subtilis* (GB03). Mol. Plant-Microbe Interact..

[98] Rhodes D., Hanson A.D. (1993). Compounds in Higher Plants. Annu. Rev. Plant Physiol. Plant Mol. Biol..

[99] Nephali L., Piater L.A., Dubery I.A., Patterson V., Huyser J., Burgess K., Tugizimana F. (2020). Biostimulants for plant growth and mitigation of abiotic stresses: A metabolomics perspective. Metabolites.

[100] Park Y.G., Mun B.G., Kang S.M., Hussain A., Shahzad R., Seo C.W., Kim A.Y., Lee S.U., Oh K.Y., Lee D.Y. (2017). *Bacillus aryabhattai* SRB02 tolerates oxidative and nitrosative stress and promotes the growth of soybean by modulating the production of phytohormones. PLoS ONE.

[101] Van Oosten M.J., Pepe O., De Pascale S., Silletti S., Maggio A. (2017). The role of biostimulants and bioeffectors as alleviators of abiotic stress in crop plants. Chem. Biol. Technol. Agric..

[102] Othibeng K., Nephali L., Myoli A., Buthelezi N., Jonker W., Huyser J., Tugizimana F. (2022). Metabolic Circuits in Sap Extracts Reflect the Effects of a Microbial Biostimulant on Maize Metabolism under Drought Conditions. Plants.

[103] Hasin Y., Seldin M., Lusis A. (2017). Multi-omics approaches to disease. Genome Biol..

[104] Baek D., Rokibuzzaman M., Khan A., Kim M.C., Park H.J., Yun D.J., Chung Y.R. (2020). Plant-Growth Promoting *Bacillus oryzicola* YC7007 Modulates Stress-Response Gene Expression and Provides Protection from Salt Stress. Front. Plant Sci..

[105] Chauhan P.S., Lata C., Tiwari S., Chauhan A.S., Mishra S.K., Agrawal L., Chakrabarty D., Nautiyal C.S. (2019). Transcriptional alterations reveal *Bacillus amyloliquefaciens*-rice cooperation under salt stress. Sci. Rep..

[106] Subramanian S., Souleimanov A., Smith D.L. (2021). Thuricin17 Production and Proteome Differences in *Bacillus thuringiensis* NEB17 Cell-Free Supernatant under NaCl Stress. Front. Sustain. Food Syst..

[107] Hall R.D., D’Auria J.C., Silva Ferreira A.C., Gibon Y., Kruszka D., Mishra P., van de Zedde R. (2022). High-throughput plant phenotyping: A role for metabolomics?. Trends Plant Sci..

[108] van Dam N.M., Bouwmeester H.J. (2016). Metabolomics in the Rhizosphere: Tapping into Belowground Chemical Communication. Trends Plant Sci..

[109] Comolli L.R. (2014). Intra- and inter-species interactions in microbial communities. Front. Microbiol..

[110] Pascale A., Proietti S., Pantelides I.S., Stringlis I.A. (2020). Modulation of the Root Microbiome by Plant Molecules: The Basis for Targeted Disease Suppression and Plant Growth Promotion. Front. Plant Sci..

[111] Vishwakarma K., Kumar N., Shandilya C., Mohapatra S., Bhayana S., Varma A. (2020). Revisiting Plant–Microbe Interactions and Microbial Consortia Application for Enhancing Sustainable Agriculture: A Review. Front. Microbiol..

[112] Yin C., Casa Vargas J.M., Schlatter D.C., Hagerty C.H., Hulbert S.H., Paulitz T.C. (2021). Rhizosphere community selection reveals bacteria associated with reduced root disease. Microbiome.

[113] Nyholm L., Koziol A., Marcos S., Botnen A.B., Aizpurua O., Gopalakrishnan S., Limborg M.T., Gilbert M.T.P., Alberdi A. (2020). Holo-Omics: Integrated Host-Microbiota Multi-omics for Basic and Applied Biological Research. iScience.

[114] Li D., Carr G., Zhang Y., Williams D.E., Amlani A., Bottriell H., Mui A.L.F., Andersen R.J. (2011). Turnagainolides A and B, cyclic depsipeptides produced in culture by a *Bacillus* sp.: Isolation, structure elucidation, and synthesis. J. Nat. Prod..

[115] Tareq F.S., Shin H.J. (2017). Bacilotetrins A and B, Anti-Staphylococcal Cyclic-Lipotetrapeptides from a Marine-Derived *Bacillus subtilis*. J. Nat. Prod..

[116] Ravu R.R., Jacob M.R., Chen X., Wang M., Nasrin S., Kloepper J.W., Liles M.R., Mead D.A., Khan I.A., Li X.C. (2015). Bacillusin A, an antibacterial macrodiolide from *Bacillus amyloliquefaciens* AP183. J. Nat. Prod..

[117] Tareq F.S., Lee M.A., Lee H.S., Lee J.S., Lee Y.J., Shin H.J. (2014). Gageostatins A-C, antimicrobial linear lipopeptides from a marine *Bacillus subtilis*. Mar. Drugs.

[118] Romero-Tabarez M., Jansen R., Sylla M., Lünsdorf H., Häußler S., Santosa D.A., Timmis K.N., Molinari G. (2006). 7-O-malonyl macrolactin A, a new macrolactin antibiotic from *Bacillus subtilis* active against methicillin-resistant Staphylococcus aureus, Vancomycin-resistant enterococci, and a small-colony variant of Burkholderia cepacia. Antimicrob. Agents Chemother..

[119] Ma Z., Hu J. (2018). Plipastatin A1 produced by a marine sediment-derived *Bacillus amyloliquefaciens* SH-B74 contributes to the control of gray mold disease in tomato. 3 Biotech.

[120] Chakraborty K., Thilakan B., Raola V.K. (2017). Antimicrobial polyketide furanoterpenoids from seaweed-associated heterotrophic bacterium *Bacillus subtilis* MTCC 10403. Phytochemistry.

[121] Xie C.L., Xia J.M., Su R.Q., Li J., Liu Y., Yang X.W., Yang Q. (2018). Bacilsubteramide A, a new indole alkaloid, from the deep-sea-derived *Bacillus subterraneus* 11593. Nat. Prod. Res..

[122] Pinzón-Espinosa A., Martinez-Matamoros D., Castellanos L., Duque C., Rodríguez J., Jiménez C., Ramos F.A. (2017). Cereusitin A, a cyclic tetrapeptide from a *Bacillus cereus* strain isolated from the soft coral Antillogorgia (syn. Pseudopterogorgia) elisabethae. Tetrahedron Lett..

[123] Jin J., Lao J., Zhou R., He W., Qin Y., Zhong C., Xie J., Liu H., Wan D., Zhang S. (2018). Simultaneous identification and dynamic analysis of saccharides during steam processing of rhizomes of Polygonatum cyrtonema by HPLC–QTOF–MS/MS. Molecules.

[124] Anjum K., Bi H., Chai W., Lian X.Y., Zhang Z. (2018). Antiglioma pseurotin A from marine *Bacillus* sp. FS8D regulating tumour metabolic enzymes. Nat. Prod. Res..

[125] Le Marrec C., Hyronimus B., Bressollier P., Verneuil B., Urdaci M.C. (2000). Biochemical and genetic characterization of coagulin, a new antilisterial bacteriocin in the pediocin family of bacteriocins, produced by *Bacillus coagulans* I4. Appl. Environ. Microbiol..

[126] Kamoun F., Mejdoub H., Aouissaoui H., Reinbolt J., Hammami A., Jaoua S. (2005). Purification, amino acid sequence and characterization of Bacthuricin F4, a new bacteriocin produced by *Bacillus thuringiensis*. J. Appl. Microbiol..

[127] Bizani D., Dominguez A.P.M., Brandelli A. (2005). Purification and partial chemical characterization of the antimicrobial peptide cerein 8A. Lett. Appl. Microbiol..

[128] Lisboa M.P., Bonatto D., Bizani D., Henriques J.A.P., Brandelli A. (2006). Characterization of a bacteriocin-like substance produced by *Bacillus amyloliquefaciens* isolated from the Brazilian Atlantic forest. Int. Microbiol..

[129] Chehimi S., Delalande F., Sablé S., Hajlaoui M.R., Van Dorsselaer A., Limam F., Pons A.M. (2007). Purification and partial amino acid sequence of thuricin S, a new anti-*Listeria* bacteriocin from *Bacillus thuringiensis*. Can. J. Microbiol..

[130] Kalinovskaya N.I., Kuznetsova T.A., Ivanova E.P., Voinov V.G., Huth F., Laatsch H. (2002). Characterization of Surfactin-like Cyclic Depsipeptides Synthesized by *Bacillus pumilus* from Ascidian Halocynthia aurantium. Mar. Biotechnol..

[131] Steinborn G., Hajirezaei M.R., Hofemeister J. (2005). bac genes for recombinant bacilysin and anticapsin production in *Bacillus* host strains. Arch. Microbiol..

[132] Tamehiro N., Okamoto-Hosoya Y., Okamoto S., Ubukata M., Hamada M., Naganawa H., Ochi K. (2002). Bacilysocin, a novel phospholipid antibiotic produced by *Bacillus subtilis* 168. Antimicrob. Agents Chemother..

[133] Luzzatto-Knaan T., Melnik A.V., Dorrestein P.C. (2019). Mass Spectrometry Uncovers the Role of Surfactin as an Interspecies Recruitment Factor. ACS Chem. Biol..

[134] Watrous J., Roach P., Alexandrov T., Heath B.S., Yang J.Y., Kersten R.D., Van Der Voort M., Pogliano K., Gross H., Raaijmakers J.M. (2012). Mass spectral molecular networking of living microbial colonies. Proc. Natl. Acad. Sci. USA.

[135] Tugizimana F., Engel J., Salek R., Dubery I., Piater L., Burgess K., Doorsamy W., Paul B.S., Marwala T. (2020). The disruptive 4IR in life sciences: Metanolomics. The Disruptive Fourth Industrial Revolution.

[136] Dodds J.N., Baker E.S. (2019). Ion Mobility Spectrometry: Fundamental Concepts, Instrumentation, Applications, and the Road Ahead. J. Am. Soc. Mass Spectrom..

[137] Armenta S., Esteve-Turrillas F.A., Alcalà M. (2020). Analysis of hazardous chemicals by “stand alone” drift tube ion mobility spectrometry: A review. Anal. Methods.

[138] Ratiu I.A., Bocos-Bintintan V., Patrut A., Moll V.H., Turner M., Thomas C.L.P. (2017). Discrimination of bacteria by rapid sensing their metabolic volatiles using an aspiration-type ion mobility spectrometer (a-IMS) and gas chromatography-mass spectrometry GC-MS. Anal. Chim. Acta.

[139] Quinn R.A., Nothias L.F., Vining O., Meehan M., Esquenazi E., Dorrestein P.C. (2017). Molecular Networking As a Drug Discovery, Drug Metabolism, and Precision Medicine Strategy. Trends Pharmacol. Sci..

[140] Rogers S., Ong C.W., Wandy J., Ernst M., Ridder L., Van Der Hooft J.J.J. (2019). Deciphering complex metabolite mixtures by unsupervised and supervised substructure discovery and semi-automated annotation from MS/MS spectra. Faraday Discuss..

[141] Nothias L.F., Petras D., Schmid R., Dührkop K., Rainer J., Sarvepalli A., Protsyuk I., Ernst M., Tsugawa H., Fleischauer M. (2020). Feature-based molecular networking in the GNPS analysis environment. Nat. Methods.

[142] Wang T., Lu Q., Sun C., Lukianov D., Osterman I.A., Sergiev P.V., Dontsova O.A., Hu X., You X., Liu S. (2020). Hetiamacin E and F, New amicoumacin antibiotics from *Bacillus subtilis* PJS using MS/MS-based molecular networking. Molecules.

[143] Purves K., Macintyre L., Brennan D., Hreggviðsson G., Kuttner E., Ásgeirsdóttir M.E., Young L.C., Green D.H., Edrada-Ebel R., Duncan K.R. (2016). Using molecular networking for microbial secondary metabolite bioprospecting. Metabolites.

[144] Meyer H., Weidmann H., Lalk M. (2013). Methodological approaches to help unravel the intracellular metabolome of *Bacillus subtilis*. Microb. Cell Fact..

[145] Mondol M.A.M., Shin H.J., Islam M.T. (2013). Diversity of secondary metabolites from marine bacillus species: Chemistry and biological activity. Mar. Drugs.

[146] Shahid I., Han J., Hanooq S., Malik K.A., Borchers C.H., Mehnaz S. (2021). Profiling of Metabolites of *Bacillus* spp. and Their Application in Sustainable Plant Growth Promotion and Biocontrol. Front. Sustain. Food Syst..

[147] Nigris S., Baldan E., Tondello A., Zanella F., Vitulo N., Favaro G., Guidolin V., Bordin N., Telatin A., Barizza E. (2018). Biocontrol traits of *Bacillus licheniformis* GL174, a culturable endophyte of Vitis vinifera cv. Glera 06 Biological Sciences 0604 Genetics 06 Biological Sciences 0607 Plant Biology 06 Biological Sciences 0605 Microbiology. BMC Microbiol..

[148] Miljaković D., Marinković J., Balešević-Tubić S. (2020). The significance of *Bacillus* spp. in disease suppression and growth promotion of field and vegetable crops. Microorganisms.

[149] Tran C., Cock I.E., Chen X., Feng Y. (2022). Antimicrobial *Bacillus*: Metabolites and Their Mode of Action. Antibiotics.

[150] Mhlongo M.I., Piater L.A., Madala N.E., Labuschagne N., Dubery I.A., Hall R.D. (2018). The chemistry of plant–microbe interactions in the rhizosphere and the potential for metabolomics to reveal signaling related to defense priming and induced systemic resistance. Front. Plant Sci..

[151] Andrić S., Meyer T., Ongena M. (2020). *Bacillus* Responses to Plant-Associated Fungal and Bacterial Communities. Front. Microbiol..

[152] Venturi V., Bez C. (2021). A call to arms for cell–cell interactions between bacteria in the plant microbiome. Trends Plant Sci..

[153] Korenblum E., Dong Y., Szymanski J., Panda S., Jozwiak A., Massalha H., Meir S., Rogachev I., Aharoni A. (2020). Rhizosphere microbiome mediates systemic root metabolite exudation by root-to-root signaling. Proc. Natl. Acad. Sci. USA.

[154] Wen T., Zhao M., Liu T., Huang Q., Yuan J., Shen Q. (2020). High abundance of *Ralstonia solanacearum* changed tomato rhizosphere microbiome and metabolome. BMC Plant Biol..

[155] Andrić S., Meyer T., Rigolet A., Prigent-Combaret C., Höfte M., Balleux G., Steels S., Hoff G., De Mot R., McCann A. (2021). Lipopeptide Interplay Mediates Molecular Interactions between Soil Bacilli and Pseudomonads. Microbiol. Spectr..

[156] Kang W.S., Chen L.J.L.J., Wang Y.Y., Zhu X.F., Liu X.Y., Fan H., Duan Y.X. (2020). *Bacillus* simplex treatment promotes soybean defence against soybean cyst nematodes: A metabolomics study using GC-MS. PLoS ONE.

[157] Carlson R., Tugizimana F., Steenkamp P.A., Dubery I.A., Hassen A.I., Labuschagne N. (2020). Rhizobacteria-induced systemic resilience in *Sorghum bicolor* (L.) moench against Fusarium pseudograminearum crown rot under drought stress conditions. Biol. Control.

